# Finishing monkeypox genomes from short reads: assembly analysis and a neural network method

**DOI:** 10.1186/s12864-016-2826-8

**Published:** 2016-08-31

**Authors:** Kun Zhao, Robert M. Wohlhueter, Yu Li

**Affiliations:** 1 Office of Infectious Diseases, Centers for Disease Control and Prevention, Atlanta, 30333 USA; 2 Department of Chemistry, Georgia State University, Atlanta, 30303 USA; 3 Poxvirus and Rabies Branch, Division of High Consequence Pathogens and Pathology, National Center for Emerging and Zoonotic Infectious Diseases, Centers for Disease Control and Prevention, Atlanta, 30333 USA

**Keywords:** Poxvirus, Neural Network, *de novo* Assembly, Whole-genome sequencing, Repetitive sequence, Gap filling, Graph, Public health

## Abstract

**Background:**

Poxviruses constitute one of the largest and most complex animal virus families known. The notorious smallpox disease has been eradicated and the virus contained, but its simian sister, monkeypox is an emerging, untreatable infectious disease, killing 1 to 10 % of its human victims. In the case of poxviruses, the emergence of monkeypox outbreaks in humans and the need to monitor potential malicious release of smallpox virus requires development of methods for rapid virus identification. Whole-genome sequencing (WGS) is an emergent technology with increasing application to the diagnosis of diseases and the identification of outbreak pathogens. But “finishing” such a genome is a laborious and time-consuming process, not easily automated. To date the large, complete poxvirus genomes have not been studied comprehensively in terms of applying WGS techniques and evaluating genome assembly algorithms.

**Results:**

To explore the limitations to finishing a poxvirus genome from short reads, we first analyze the repetitive regions in a monkeypox genome and evaluate genome assembly on the simulated reads. We also report on procedures and insights relevant to the assembly (from realistically short reads) of genomes. Finally, we propose a neural network method (namely Neural-KSP) to “finish” the process by closing gaps remaining after conventional assembly, as the final stage in a protocol to elucidate clinical poxvirus genomic sequences.

**Conclusions:**

The protocol may prove useful in any clinical viral isolate (regardless if a reference-strain sequence is available) and especially useful in genomes confounded by many global and local repetitive sequences embedded in them. This work highlights the feasibility of finishing real, complex genomes by systematically analyzing genetic characteristics, thus remedying existing assembly shortcomings with a neural network method. Such finished sequences may enable clinicians to track genetic distance between viral isolates that provides a powerful epidemiological tool.

## Background

Poxviruses are one of the largest and most complex animal virus family known [1]; the subfamily chordopoxvirinae comprises at least eight genera (*orthopoxvirus*, *capripoxvirus*, *leporipoxvirus*, *suipoxvirus*, *parapoxvirus*, *yatapoxvirus*, *avipoxvirus*, and *molluscipoxvirus*). The orthopoxvirus variola virus (VARV; a.k.a. smallpox) was wiped out of a human history in the 1970s, thanks to the success of vaccination. Smallpox eradication counts as one of the greatest triumphs of modern medicine. Before the eradication, smallpox caused from 30 to 35 % case-fatality rates (CFRs). It is a highly contagious and strictly human disease, which caused an estimated 300–500 million deaths during the 20th century alone [2].

Human smallpox has a simian sister, monkeypox virus (MPXV). This virus, also of the *orthopox* genus, causes an endemic disease, first recognized in Africa in 1970, but with an outbreak in 2003 in the United States, that was traced to imported monkeypox virus-infected West African rodents [3]. MPXV causes smallpox-like disease in humans; in Africa the disease typically kills between 1 and 10 % of its human victims [4]. Smallpox vaccination is known to lower the risk of contracting monkeypox, but there is no specific immunization against monkeypox *per se*. Due to a declining immunity to orthopoxviruses in the general population, there is a risk that monkeypox might emerge as a significant human pathogen.

With better timeliness and accuracy, whole-genome sequencing (WGS) holds promise of revolutionizing health surveillance systems and possibly of resolving many current limitations associated with poor pathogen discrimination [5–7]. Genomic information offers a profound increase in the resolution of pathogen type, enabling possible identification of geographic origin and whether the agent is previously known or represents a novel mutant. Mature data processing methodologies developed to address the decades-long preponderance of Sanger sequencing data are not always adaptable to the characteristics of WGS sequencers, which have produced prodigious data, but only over a period of ten years [8]. Bioinformatician is turning the computational challenges in the WGS technology to opportunities for developing new algorithms, or improving efficiencies of existing ones as to processing raw data into medically useful sequences.

WGS technology produces millions of short sequence fragments (“reads”). The reads are ordered and combined into longer sequences called “contigs” (a.k.a “assemblies”). Finally the contigs are ordered to produce a complete genomic sequence. This highest level ordering can often exploit known genomic reference in agents, like poxvirus, for which there are reliable genomic sequences available. Of course, this entire assembly process, whether *de novo* or against a known reference, demands appropriate and efficient computational algorithms.

Given an accurate genomic sequence, it becomes possible to deduce unique SNP/Indel profiles to facilitate quick-and-easy field diagnosis of a particular strain. Such unambiguous diagnosis, in turn, triggers a range of epidemiological tracking and public health surveillance and control systems.

At a theoretical level, the new genomic information supports phylogenetic clustering analysis, which can place an outbreak strain into the broader context of the poxvirus “family tree”. Such analysis often revels the ultimate geographic origin of a new strain, a rough chronology of its emergence, and the plausibility that it is a cross-over from a xenobiotic strain, like monkeypox. Both tactical and theoretical motives have served to put pathogen genomic sequencing high on the priority lists of public health agencies, such as the CDC.

A definition of “high-quality” has been promulgated by Genome Assembly Gold-Standard Evaluations (GAGE) [9, 10] and applied in two competitions for quality assembly: “Assemblathon” [11, 12] and the “*De Novo* Genome Assembly Assessment Project” (dnGASP) [13]. These efforts evaluated the contigs generated by popular assembler software. Assembly of the complex poxvirus genomes were poorly represented in the competitions: only the assembly of swinepox genome was evaluated, and that only from simulated data with fixed read-lengths of 75 (paired-end) [14]. It is well recognized that good performance of assembler software on simulated data may not reflect its performance on real data, which often include gaps, inverts, and rearrangements, usually generated from shorter reads. Moreover, these evaluations were focused on the computational times and ignored the peculiarities of real, complex genomes.

For example, in the long poxvirus genome, gaps (in reference to related poxviruses) are common, and may be of biological significance [3, 15–18]. Moreover, poxvirus genomes are known to contain “inverted terminal repeats” (ITR’s) – longer or shorter, but comprising as much as 1 % of the genome, and prone to hair-pin loop-outs [3, 18]. ITR’s have traditionally confounded poxvirus sequencers, who have usually simply ignored them in published sequences. Regions of ambiguity can be (and have sometimes been) addressed by laborious, “manual” sequencing of PCR amplicons [19], but these approaches are costly and time-consuming, and thus defeat the main advantages of WGS.

To our knowledge, no one has systematically reported on procedures or insights relevant to the assembly from short reads of genomes with peculiarities like those in monkeypox, although numerous papers have compared quality of assembly for different types of sequencing data, such as long reads. The general computational challenge to the assembly of genomes with repetitive sequences has been addressed [13]. But the general case seems to oversimplify the particular problems of repetitive regions in the poxvirus genome which has inverted terminal repeat (ITR) at each end of the genome with a size range of 2 to 12Kbp [2, 15, 16, 18–21]. The ITR region contains additional local repeats within global repeats, which pose a special challenge to the assembly problem. This unique feature is presented in the entire family of poxviruses, comprising a large number of species-specific viruses, infecting a long list of mammalian species, and of significant agricultural and wild-life impact. The focus of the study was on the MPVX short reads (such as Hi-Seq/Mi-Seq), because short-read sequencers have become a major diagnostics tool for epidemiologists, providing fast results as needed during outbreak investigations. We are actively pursuing evaluations of long-read methods, such as PacBio, with a view to obtaining a single contig which covers the entire genome. So far, we have not attained this goal, but feel the present study is a worthwhile, interim report.

Our work with poxvirus genomic assembly posed many questions. Is it even realistic to expect that *de novo* assembly algorithm can arrive at a single contig covering the whole poxvirus genome, especially from the short reads available from “next generation sequencing” technology (NGS)? If an unassembled region is observed, how do we decide whether the problem lies with the bioinformatics tools, or with sequencing chemistry? Can we simply ignore such problems, or do they reflect biologically significant characteristics of the genome? Methodological corollaries to these questions are: Can obtaining additional short-read data improve the quality of assembly? If yes, how much additional data is needed? Are there technical compromises which adequately cope with the problems while still supporting robust public health surveillance requirements?

To answer these questions, we first analyze a reference (e.g., monkeypox) genome. We then generate simulated WGS reads from the reference genome. The *de novo* contigs derived from new sequences are evaluated by comparing to the reference genome, which process identifies mis-assembly and gap regions. This strategy not only allows us to deduce a high-quality genomic sequence for the strain of the virus under study, but also allows us to understand the limitations of the assembler algorithms, and hopefully to remedy them.

We then show how gap-filling of the genome can be converted into the *all k shortest path* (KSP) problem. Finally we propose a neural network method (namely Neural-KSP) to show that it is possible to finish a monkeypox genome of a clinical sample by utilizing this method.

## Results and discussion

### Monkeypox genome, ITRs and tandem repeats

The repetitive sequences in the poxvirus genome have been reported previously [3, 18, 20, 21], but they have not been systematically correlated to the WGS analysis from short reads. In this section, we focus first on an analysis of a known monkeypox genome.

**Fig. 1:**
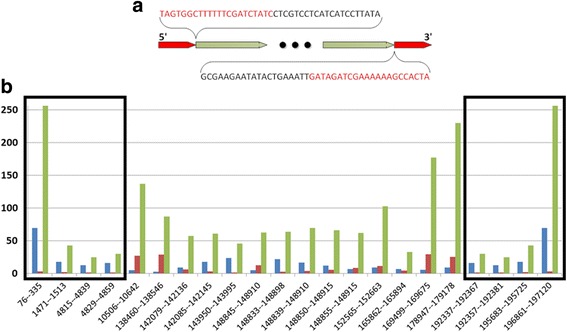
**a** A simple illustration of monkeypox genome (accession number: DQ011154) with ITR regions highlighted in *red.* The *red* sequences (nucleotide positions: 1-6477 and 190719-197195) are inverted with respect to each other. **b** *x-axis*: the tandem repeat locations in monkeypox. *y-axis*: their period size (*blue bars*), copy size (*red bars*) and the length (*green bars*) of the tandem repeat region. *Two black boxes* highlight tandem repeats within the ITR region. Tandem repeats were calculated by Tandem Repeat Finder [23]

Repeats, which can cause breaks in contigs and thus mis-assembly, can be divided into two groups: global and local. A global repeat is defined as a long sequence which is duplicated throughout the genome [22]. The ITR repeat in the monkeypox genome is one typical example of the global repeat [22]. For example, the sequence of the monkeypox genome deposited in GenBank (Accession No. DQ011154) has a length of 197,195 bp with 206 annotated coding sequences (CDS). The two ends of the genome (ITR regions) are identical, but inverted, with a length of 6,477 bp. The many coding sequences which connect the two ends are abbreviated as black dots in Fig. 1a, while the red sequences are inverted with respect to each other.

In contrast to global repeats, the local repeat contains a simple sequence, which is duplicated in tandem many times. In the monkeypox genome, local tandem repeats are found both within the ITR regions and outside these regions; that is, local repeats may be nested within global repeats. Specifically, there are total 22 tandem repeat regions [23]. Four of them are in the ITR region. As shown in Fig. 1b, the full lengths of each tandem repeat varies from 24 to 250 bp, the unique period size are from 3 to 70 bp, and the copy sizes are from 2 to 30. These tandem repeat regions seem to be spread more-or-less randomly in the genome. This genetic characteristic of the MPXV genome leads, mathematically speaking, to the branching path problem, and usually breaks the assembly [24].

### *De novo* assembly of simulated monkeypox data

With the repeat information in hand, we simulated the Illumina**®** intrument’s (Hi-Seq 2500) paired-end reads using two different lengths: 100 and 250 bp, and at various coverage depths (e.g., from 5X to 10,000X). Reads were simulated using the fastqSimulate tool in Celera Assembler (version 8.1). Three types of errors at a level of 1 % were taken into consideration while doing the simulation; namely mistmatch, insertion and deletion [25]. The reasons for choosing these read lengths are: 100 bp covers the longest period size (70 bp, as noted above), while 250 bp is approximately the longest tandem repeat in monkeypox genome. These two lengths are supported by a standard Illumina NGS techniques (100 bp for “Hi-Seq” and 250 bp for “Mi-Seq”).

With these simulations, we are trying to address several questions: 1) Are repeats the sole Achilles’ heel of the genome assembly from short reads? 2) What is the relationships among read length, longest contig size, genome coverage, genome depth coverage? 3) What is the minimum coverage sufficient for obtaining a reasonable assembly? 4) Will more sequencing reads alleviate ambiguities in *de novo* assembly?

Results of the simulation experiments are tabulated in Table 1. For simulated 100-bp read lengths, while decreasing the total number of reads from 50 to 10K (last two rows), we observed that coverage decreased from 25X to 5X, leading to a large increase of the total number of contigs and a decrease of maximum contig length. This is simply due to insufficient sequence data. Twelve million, 100 bp-reads yields the fewest and longest contigs, which corresponds to about 90 % coverage of the genome with 100 % accuracy.

**Table 1:** Statistics for *de novo* assemblies using simulated monkeypox reads

	Read length: 100			Read length: 250		
Total Reads	Coverage	Contigs Numbers	Longest Contig (bp)	Coverage	Contigs Numbers	Longest Contig (bp)
20M	9760X	7	127K	24971X	4	135K
12M	6085X	2	183K	15213X	25	159K
7.5M	3803X	5	159K	8523X	31	169K
2M	910X	4	159K	2535X	4	184K
1M	450X	5	161K	1280X	4	168K
100K	50X	5	159K	127X	4	186K
50K	25X	7	142K	63X	1	189K
10K	5X	108	4K	12X	6	95K

A general performance pattern can be perceived: to obtain maximal contig length, there exists an optimal coverage at given read lengths for any fixed set of assembly parameters. (e.g., here we set mismatch = 0.8 and alignment = 0.9 in the CLC Bio de novo assembly for all simulated read-lengths). Although, this is the first report of simulation results based on the monkeypox genome, the phenomenon of diminishing returns with increasing sequencing effort has been reported before [14]. Thus, for any simulated read-length, collecting additional sequence data beyond this optimum does not improve assembly, but costs unproductive effort, as when we increase the number of reads from 12 to 20M, at a 100-bp read length for the example in Table 1. We do not suggest that this is a mathematically provable theorem, nor are we certain of this physical basis. But it is a consistent feature over a wide range of assembly parameter settings, generating large disparities of contig lengths (results with only one parameter set are reported here). We venture to surmise that the superfluous coverage introduces more noise to the data, in a way that confounds the random algorithm rather than contributing to finding longer contigs.

The simulations indicate that increasing the read length to 250 bp can be expected to improve the *de novo* assembly, but, of course, at a price of increasing reagent consumption and sequencing time (for example, doubling Illumina read length will lead to a doubling of the sequencing time). In Table 1, the longest contig length was achieved at 250-bp reads: 50 thousand reads provides 60X coverage, with a single contig of 189Kbp. This observation highlights a rule-of-thumb that long reads require less coverage for assembly. Regardless of simulated read length, assembly breaks in the ITR regions due to the global and local repeats.

### Recasting the assembly task into a graph-theory context

Before describing our results with real-world, monkeypox sequence data, it is useful to review briefly the concepts relating the *de novo* sequence assembly problem to graph theory. Details and examples of our specific approach are laid out in the Methods section below.

Utilizing graph methods to deal with the assembly problem has typically involved constructing an overlapping, or *de Bruijn* graph, where each node in the graph represents a short sequence fragment (either a read or k-mer) [26, 27]. If there is an overlap between two fragments (overlapping method), or if k-mers have a particular prefix and suffix (the *de Bruijn* graph method), an edge is added between the corresponding nodes. A weight is assigned to this edge, which takes into consideration the length of the overlap and possibly other factors (e.g., indels, gaps, sequencing errors, etc.). In effect, a contig can be considered as a path in the graph from an initial node to a terminal node (specifically, initial read to terminal read in an overlapping graph or initial k-mer to terminal k-mer in a *de Bruijn* graph). The intrinsic problem of deducing and ranking several possible contigs (from a data set of millions of fragments, all similar in length) which join a set of specific, overlapping, short fragments is equivalent to searching out a path (or paths) in a graph.

Velvet [28] has utilized a Dijkstra-like algorithm to search out paths in *de Bruijn* graphs. The approach confronts two of the general challenges: 1) A sequence repetition (e.g., a monkeypox ITR) represents a branch in the paths through the graph, at which the algorithm gets confused, and lacking some additional rule, the assembly process stops [24]. 2) As the number of reads increases, the complexity of the graph becomes so large that it overwhelms computation of the true path. The branch-point dilemma has been ameliorated somewhat by reporting the best path so far, before giving up in failure [29]. The complexity issue is greatly exacerbated by new generation instruments, which churn out many more, but significantly shorter, reads.

These previous efforts highlight the fact that global and local repeats are fundamental obstacles to the application of graph-theory to *de novo* assembly, because such repeats constitute branching points which terminate as broken contigs [24]. Such gaps are the major challenge to “finishing” a genome, that is, to generating a single, unbroken contig. Our approach provides a partial solution to these dilemmae by delineating multiple, “shortest” paths, that is, a few, alternative, unbroken contigs, one of which most likely represents the “true” genomic sequence.

Rather than holding out for *the* shortest path, and risking failure, we settle for the *k*-shortest paths. At branch-points caused by repetitive sequences, we pursue all possible branches, and rank the ultimate complete paths by length. To solve the k shortest path problem (KSP) we resort to neural networks.

### The all k shortest path problem (KSP) problem and a neural network method

To find all k shortest paths in a graph needs not only computation of the shortest path from the initial node to the end node, but also computation of the second, third… kth shortest paths (if available). Typically, it is solved by heuristic algorithms, such as the well-known Dijkstra's algorithm, which can quickly provide a good solution in most instances. However, as the scale of problem increases, these methods become inefficient and may consume considerable amounts of CPU time. Neural networks, which are massively parallel models, have been reported as an approach to circumvent these problems with the classical algorithms [30–33]. Continuous-time coupled neural networks in have been advocated as effective approaches [34–36] to solving shortest path (SP) problems. In these methods, decision variables *v*_*ij*_ (a.k.a., edges in a graph) are denoted as the neuron activation states. These state are described by a system of differential equations. A Lyapunov (energy) function is designed to drive each neuron to its stable state [34, 37]. Usually, the terms “neuron” and “node” were used interchangeably, though by “neuron” we imply also the system of differential equations associated with that node’s activation. Recent techniques use vertices in the graph to denote neurons [33, 38]. The neural dynamics are modeled by coupled differential equations in such a way that a smaller coupling strength (e.g., connection weights in a graph) corresponds to an individual neuron’s earlier firing time. Following excitation of the initial neuron, the signal propagates according to the graph topology and individual neural dynamics – across (a properly constructed) network, from the initial node to terminal node through all paths, including the shortest path.

One advantage of this method is that the time required to propagate of the signal (wave) is not dependent of the number of nodes in the graph, but only on the path lengths from the initial node to the terminal node [38]. It is this characteristic that addresses the second dilemma, namely, graph node-complexity inherent in NGS chemistry. To find the shortest path, one must calculate the individual node dynamics; closed-form solutions are available in system which contain only first-order linear differential equations (see [38]).

We have previously extended a neural network method to the KSP problem (namely Neural-KSP) for a graph with multiple edges and demonstrated its independence of the number of nodes and edges but on a graph topology [38]. The computational intensity of the Neural-KSP algorithm scales with the network topology -- performing better when all k shortest path lengths are small and the network is large [39].

With respect to gap-filling, we have also explored “GapFiller”, a program which has been validated on bacterial datasets and the human genome [40]. We were not able to fill the 6 gaps in Table 2. Thus, we embed the Neural-KSP method as the final stage in our in-house pipeline for calculating finished viral genomes. This laboratory has sequenced (and published) numerous poxvirus sequences; all previous, conventional methods of assembly have proven far more laborious. In the Methods section, we lay out a generalized formulation, taking into account both individual node dynamics and overall network topology. This form offers a mathematical foundation for the Neural-KSP method. Applying this pipe-lined process has enabled us to obtain a finished, monkeypox genome of length of 197Kbp, as shown below.

**Table 2:** Statistics for clinical sample

Raw data, number of reads	33,339,183
Read length before trimming	100 bp
Trimmed reads	31,012,094
Read length after trimming	97 bp
Unmapped to human	3,197,545
*de novo* contigs #	7
Maximum contig length	129,805 bp
Genome scaffold coverage	~96 %
Total number of gaps	6
Finished genome length	197,020 bp

### Application of the *de novo* assembly protocol to clinical monkeypox data

To explore the strategy for generating a finished genomic sequence from clinical data, we used an Illumina Hi-Seq**®** instrument (100 bp pair-end read) to sequence monkeypox virus isolated from a human patient. We expect that the method would generate better results when applied to Mi-Seq data, since it generates longer reads. However, due to limited timeline and resources, we have not been able to compare systematically results with Hi-Seq and Mi-Seq protocols. We hope to be able to do so in a follow-up report. Using standard assembly software, we expected multiple, short, gappy contigs. For this isolate, with a 33M-read datatset, the trimming process did not significantly reduce read length, as shown in Table 2. About 31M reads mapped to the human genome, and were excluded, leaving about 3M reads, presumably of viral origin. (That 90 % of the sequencing reads map to the human genome is a general caveat to researchers who would sequence clinical viral samples. This level of human contamination is probably typical.)

Using conventional assembler software (in this case from the CLC Genomics Workbench), these reads were assembled into 7 contigs, with a maximum contig length around 130kbp. These seven contigs were ordered against a *bona fide* monkeypox genome, leaving a draft genome with 6 gaps (length varied from several bases to a few hundred) and covering 96 % of the genome, including the ITR region at one end (the other is missing); these statistics are summarized in Table 3. The observations are similar to a previous study [41].

**Table 3:** Gap statistics for clinical sample

Estimated gap size^a^	Scaffolds^a^		Reference^b^	
	Start^c^	End^c^	Start^c^	End^c^
75	2224	2298	8682	8756
12	142573	142584	148875	148886
17	159557	159573	165859	165875
139	163216	163354	169518	169656
184	172663	172846	178965	179148
306	190706	191011	196890	END

^a^Gap size estimated by ABACAS; ^b^Accession No. DQ011154; ^c^Position within the sequence

The “finishing” job thus was to fill in six gaps comprising all together 733 bases (estimated by ABACAS), plus around 7Kbp missing ITR regions. Our strategy for doing this was to present, as input data to the Neural-KSP method, all 3.2M reads (pruned of human sequences) plus the gap-flanking sequences (as established by conventional assembly) – one run of the Neural-KSP for each of the six gaps.

**Fig. 2:**
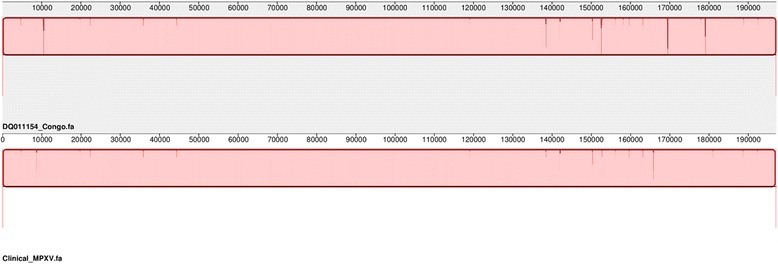
The *upper bar* depicts the reference monkeypox genome; the *lower bar* is the finished genome of the clinical isolate, assembled by the protocol described herein. The *dark red lines* within the bars track regions of multiple base differences between the two genomes, such as indels. The resolution of these charts are insufficient to reveal single nucleotide replacements (a.k.a. SNPs)

The result of this process was to fill all six gaps, producing a single contig of 197,020 bp (including ITR on both ends). The finished genome is depicted in Fig. 2, relative to the template monkeypox genome. In this particular isolate, we observed few significant genomic variations, such as indels, implying that the virus infecting the patient was genetically close to the reference strain.

## Conclusions

In summary, as final stage in a protocol to elucidate clinical poxvirus genomic sequences, we have utilized a Neural-KSP method to “finish” the process by closing gaps remaining after conventional assembly. Such finished sequences may enable clinicians to track genetic distance between viral isolates, a powerful epidemiological tool. In principle, the protocol should prove useful in any clinical viral isolate regardless if a reference-strain sequence is available. The protocol may prove especially useful in genomes confounded by many global and local repetitive sequences embedded in them.

## Methods

### Pipeline

**Fig. 3:**
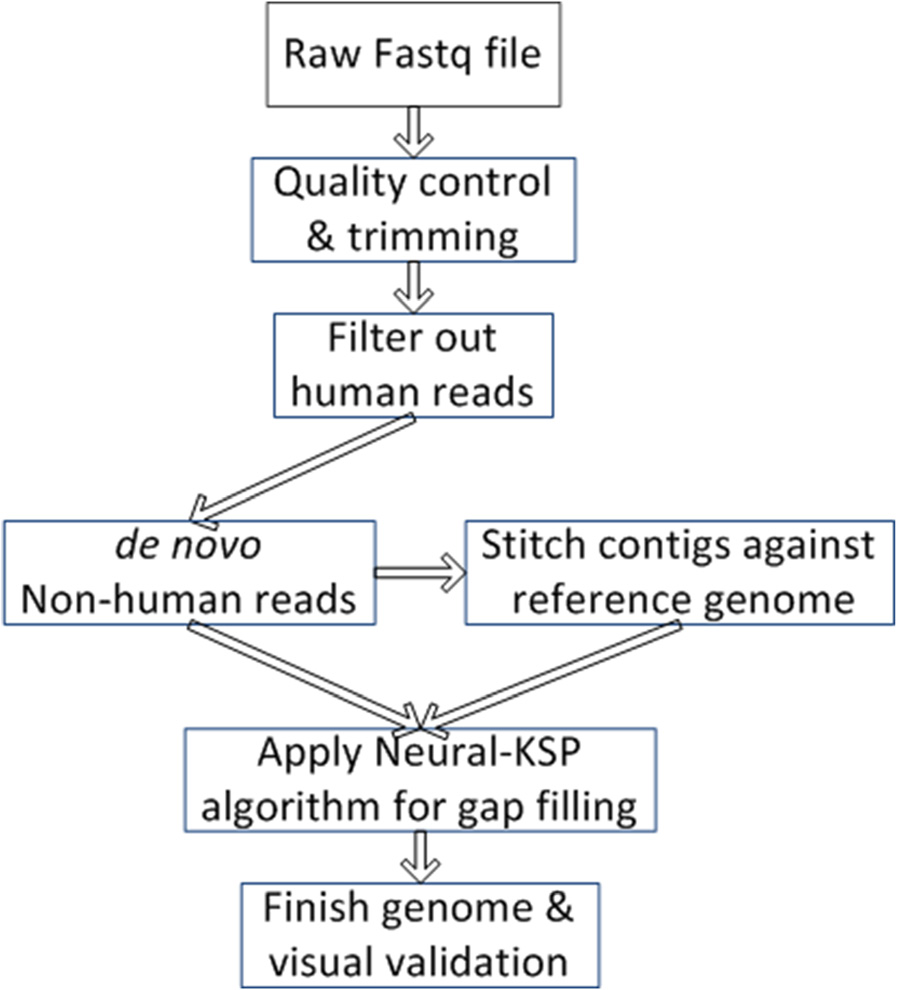
A pipeline for finishing monkeypox genome

As show in Fig. 3, the preliminary quality evaluation for each sample (FASTQ file) was generated using FASTQC. The raw data were preprocessed to remove ambiguous base calls (Ns), bases or entire reads of poor quality, and those containing adaptor sequences. After trimming, the dataset passed quality control based on “Per base sequence quality” and “Per sequence quality” scores. Next the trimmed reads were mapped to human genome to exclude any contaminating human sequences.

Thus pruned, the remaining reads were assembled *de novo* using CLC Genomics Workbench 6.5.1 (CLC Bio). We used ABACAS [42] to order de novo assembled contigs against using the information from a reference genome (a.k.a. “scaffolding”).. This tool generated a re-ordered draft genome and reported gap positions; this ordered set of contigs was used as input for the Neural-KSP method. Genome alignment and visualization were generated by MAUVE; sequence alignments were performed by BLAST and CLUSTALW. The Neural-KSP algorithm was written in-house in C++. (A prototype algorithm is available upon request). The Neural-KSP method is the last piece of the pipeline; it is summarized below.

### A general form of a coupled neural network in continuous time

We first construct a network of *n* interacting, linear/non-linear *l*-dimensional dynamical systems (neurons), and denote these neurons as *x*_*i*_ = (*x*_*i*_^1^, *x*_*i*_^2^, …, *x*_*i*_^*l*^), *i* = 1, …, *n*. We can then define a general framework for the coupled network as follows:

1 $$ {\overset{.}{x}}_i=F\left({x}_i\right)-{\displaystyle {\sum}_{j=1,j\ne i}^n{d}_{ij}(t)\tau \left({x}_j\right)} $$

where *F*(*x*_*i*_) defines each individual system, *τ*(*x*_*j*_) is an activation (sigmoidal) function, $\tau \left({x}_j\right)=1/\left(1+{e}^{\lambda \left({x}_j-\theta \right)}\right)$, which couples neurons *i* and *j*. It stipulates an excitation of the *i*th neuron by *j*th neuron, when the potential of *j*th neuron exceeds some synaptic threshold *θ*. This coupling form has been termed “fast threshold modulation” [43, 44]. *d*_*ij*_(*t*) defines the coupling strength (a.k.a weights in a graph) from neuron *i* to *j*; it should be positive and depends on time *t*. Coupling strength (an internal parameter) is defined, as explained in previous publications [32, 38, 45, 46], in such a way as to ensure that the rate of signal travel from neuron *i* to *j* is faster when the coupling strength is smaller. This form of coupling has the advantage of allowing inclusion of graphs that represent vertices as neurons.

### A neural network method for the KSP problem (Neural-KSP)

Due to the sigmoidal coupling functions, the proposed system cannot be solved analytically. A closed form solutions are available in a special case where the system contains only first-order linear differential equations (see [38]). In fact, a computer handles this problem by discretizing continuous time to discrete time. Calculation of one specific example in discrete time, as well as pseudo-code, can be found in our description of the algorithm [39], which is a discrete counterpart with respect to integrating equation 1 in a continuous time. We have evaluated the performance of the Neural-KSP algorithm for calculating all k shortest paths on a network data [33, 38, 39].

Here we describe in pseudo-code our Neural-KSP algorithm to find the all k shortest paths in an acyclic graph. It is applicable to both directed and undirected graphs so long as the graph remains loopless. Given a connected graph *G* = (*V*, *E*), let *V* denote the set of neurons (or nodes) and *E* denote the set of connections between any two neurons. *F*(*x*_*i*_) in equation 1 models the dynamics of neuron (or node) *i* and *d*_*ij*_ defines the weight between neuron *i* and *j*. In a pseudo-code, we use *d*_*ij*_ and w interchangeably. If neuron *i* excites neuron *j*, we say neuron *j* is a downstream neuron of *i*.

**Input:** An acyclic graph with or without multiple edges, designated initial and terminal neurons, and the value of k.

**Output:** all k shortest paths.

**begin:**

- **1. [initialize the network]**
  Excite the initial neuron. c = 0 (c represents the *c*th shortest path); w = 0 (w records a weight on an edge).
- **2. [track individual neuron’s excitation status at a discrete time]**
  Search minimal weighted outgoing edge(s) for the excited neuron(s).
  Record the weight as w.
  Track current excitation state of each neuron. (Please see references [38] for a calculation example.)
  If current neurons are being excited for a first time; then go to **3**.
  Elseif current neuron is in an excited state but not for the first time, track the excitation information of this neuron and attach all outgoing edges back to the neuron; then go to **3**.
  Elseif current neurons are not being excited and have not been excited yet, do nothing.
- **3. [compute the network dynamics]**
  Excite downstream neuron(s).
  - If terminal neuron arrived.
    - Track the path(s) and set c = c + 1;
    - If c = k; then go to **4**;
  - Else set all outgoing edges’ weights of the excited neurons minus w, and then go to **2**.
- **4. [end]**

### Representing gap-filling as a KSP problem

**Fig. 4:**
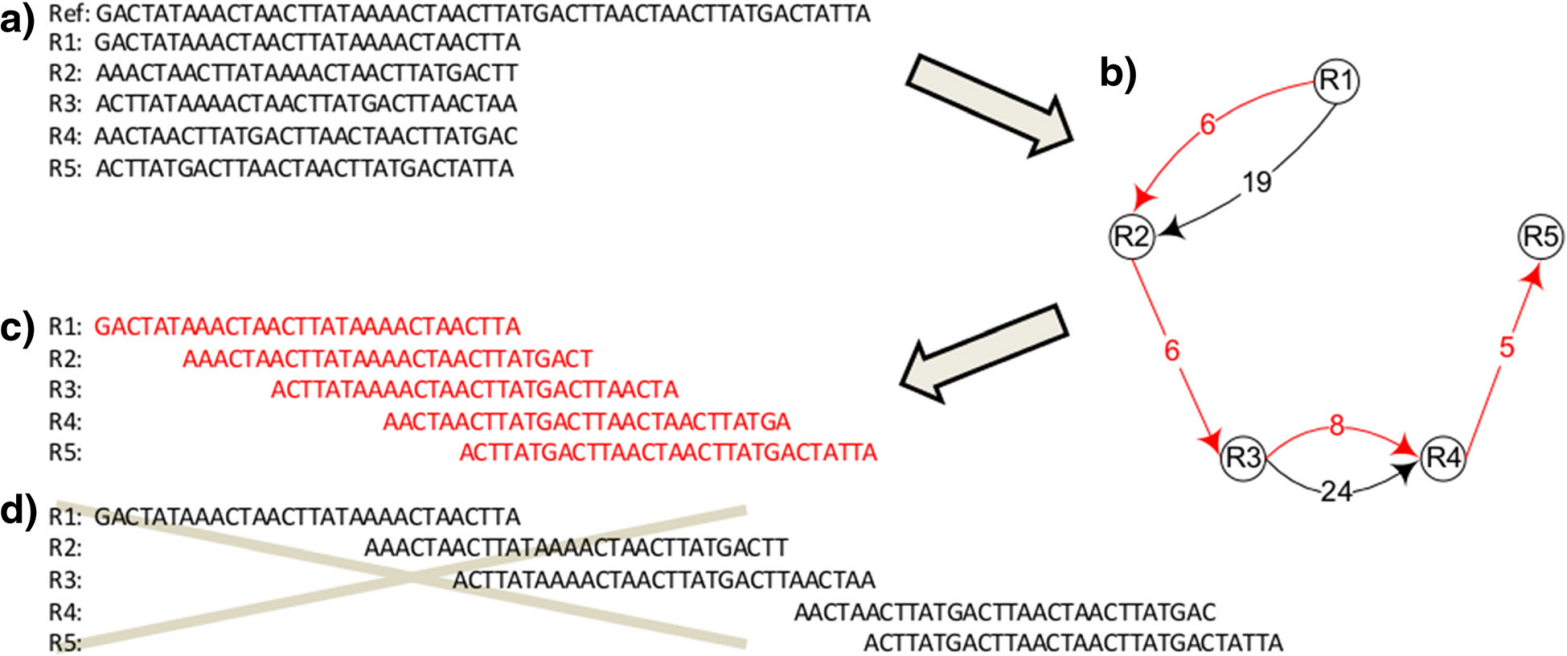
Predicting DNA sequence correctly from the shortest path [39]. **a** A reference sequence ("Ref"), positions 4809 to 4863, is taken from a published monkeypox genome (accession number: DQ011154). Sequences R1 to R5 were assumed as short fragments obtained experimentally. **b** An example of an overlapping graph in which each node represents an individual sequence (R1 to R5). An edge is added and its corresponding weight is the non-overlapping nucleotide count between any two overlapping sequences. **c** The path in *red* indicates that the sequence was assembled correctly. **d** The path in *black* indicates that the sequence was assembled incorrectly

Here we present two simple examples to show how to convert the gap-filling task into KSP problem. Let’s start with (what we believe to be) an accurate, genomic sequence of monkeypox virus (as published by the National Center of Bioinformatics, NCBI accession number DQ011154). From it, we have selected a 55 nucleotide region (positions 4809 to 4864; “Ref” in Fig. 4a), and extracted from that region five short-read sequences (“R1” to “R5”), simulating experimental data (in this case, we are mimicking data generated by the Illumina® instrument). Each short-read-sequence has 30 nucleotides. For simplicity, we assume the following: 1. R1 to R5 are randomly sampled from the reference region (“Ref”); 2. The sequenced nucleotides harbor no errors, mutations, insertions or deletions; 3. The genome sequence starts from R1 and ends at R5; 4. One wants to use as many of these short fragments as possible. These assumptions are not mandatory for the method, but facilitate illustration.

The goal is to infer the correct sequence assembly from fragments R1 to R5. Based on the non-overlapping lengths, we can build a graph in Fig. 4b. In this figure, the edge from R1 to R2, with a weight of six corresponds to six non-overlapping nucleotides in the alignment of R1 and R2 in Fig. 4c. Since the alignment is not unique, we can align them alternatively, as in 4D. The path in red corresponds to the shortest path using all fragments in Fig. 4. In this case, we have obtained the “correct” assembly by calculating shortest path.

**Fig. 5:**
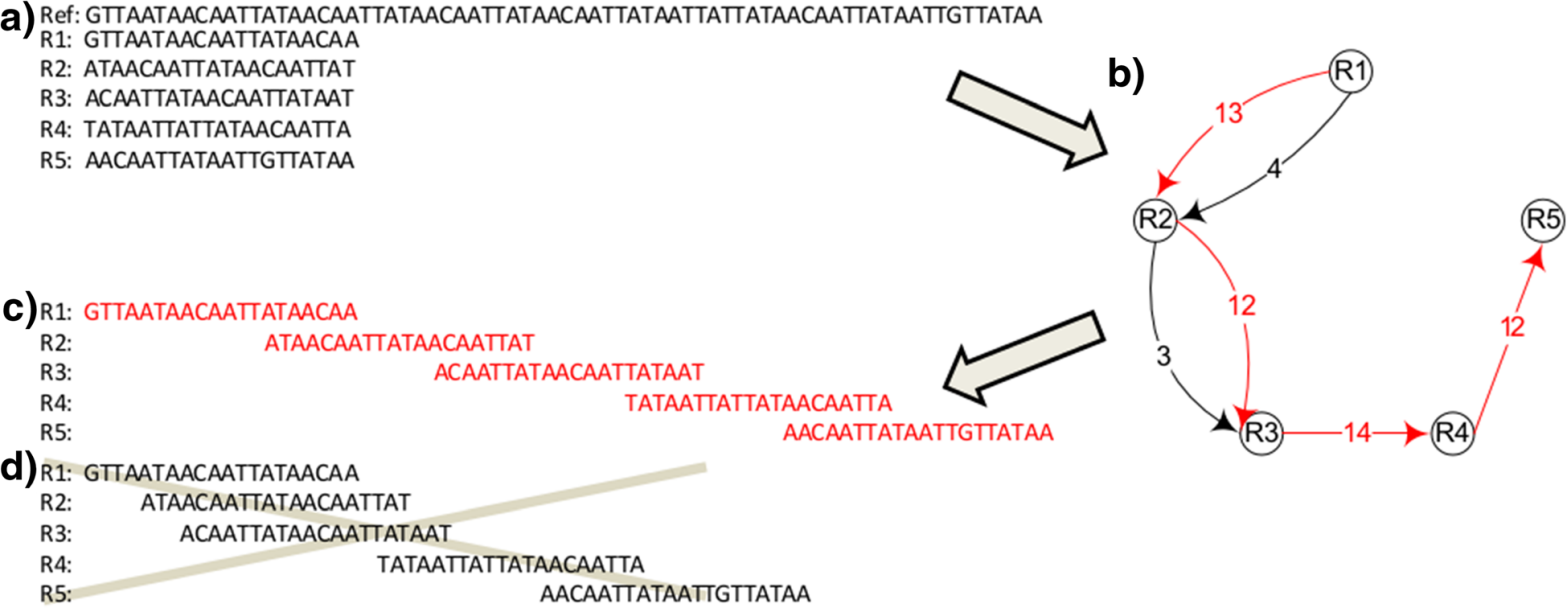
A sequence which cannot be estimated correctly based on the shortest path [39]. **a** A reference sequence ("Ref"), positions 142075 to 142145, is from a published monkeypox genome (accession number: DQ011154). Sequences R1 to R5 were assumed as short fragments obtained experimentally. **b** An example of an overlapping graph in which each node represents an individual sequence (R1 to R5). An edge is added and its corresponding weight is the non-overlapping nucleotide count between any two overlapping sequences. **c** The path in *red* indicates that the sequence was assembled correctly. **d** The path in *black* indicates that the sequence was assembled incorrectly

However, we have been able to find other regions in the reference genome for which this is not the case. For example, extracting five simulated reads from another region of the same published sequence (positions 142075 to 142145), and using them to build an analogous graph (depicted in Fig. 5b), we find that the shortest path (Fig. 5d) does not reveal the “correct” sequence assembly. Here one must calculate all k shortest paths, among which is the “correct sequence”. This is where the Neural-KSP algorithm comes in: by performing a calculation (as in ref [39]) one does obtain the “correct” DNA sequence *as one of* the top k shortest path.

Despite the advantages of the neural network to enumerate all possible paths, to decide which path (even among a few shortest k-paths) is the correct sequence is not trivial. We recommend setting k = 20 for initial gap filling (based on our observations of finishing the clinical data in Table 2). We make two assumptions to guide choice among these 20 shortest paths. First, that there is only one correct sequence; second, that the genome is sequenced randomly, so that there is no coverage bias toward some regions. Then, after mapping all reads back onto the regions to be filled, we accept as the best, filled sequence (or path) that one whose coverage is closest to the average coverage over the genome.

### Running time

As implied in Fig. 3, overall running time for finishing a genome depends on a sequential process of a few tools, including CLC Bio (genome assembly and mapping) and ABACAS (genome scaffolding), as preprocessing steps, before the application of the Neural-KSP method. On an IBM laptop equipped with 1.83 Mhz Intel CPU and 4GB RAM and running Windows 7, for the clinical sample described here, the most time-consuming step is to filter out the human reads (an ~3 billion basepair genome). This filtering takes CLC Bio ~5 h, plus 6 min to assemble the remaining, non-human reads. The ABACAS programs runs in less 1 min. The Neural-KSP method has been tested on a simulated graph with 1 million nodes and 2 million edges, for which it uses 42 s (an average of 10 runs), compared with 142 s of a Dijkstra-based algorithm [38, 39]. On the actual clinical data set, it ran in 90 s. Accordingly, our current efforts at shortening to overall analysis time is focused on parallelization of the human read filtering step.
